# Integrating macro- and meso-level perspectives on school nutrition programs in Dubai: a joint display mixed methods integration study

**DOI:** 10.3389/fpubh.2026.1915072

**Published:** 2026-10-06

**Authors:** Reem AlGurg, Farah Otaki, Khadija Albloushi, Ghadah A. Al-Sharif, Nour Abu Mahfouz, Agnes Paulus, Amar Khamis, Mohamad Alameddine

**Affiliations:** 1 Strategy and Institutional Excellence, Mohammed Bin Rashid University of Medicine and Health Sciences (MBRU), Dubai, United Arab Emirates; 2 College of Medicine, Mohammed Bin Rashid University of Medicine and Health Sciences (MBRU), Dubai, United Arab Emirates; 3 Department of Health Services Research, Care and Public Health Research Institute (CAPHRI), Faculty of Health, Medicine, and Life Sciences (FHML), Maastricht University, Maastricht, Netherlands; 4 Graduate Medical Education, Mohammed Bin Rashid University of Medicine and Health Sciences (MBRU), Dubai, United Arab Emirates; 5 Faculty of Health, Medicine, and Life Sciences (FHML), School of Health Professions Education (SHE), Maastricht University, Maastricht, Netherlands; 6 Hamdan Bin Mohammed College of Dental Medicine, Mohammed Bin Rashid University of Medicine and Health Sciences (MBRU), Dubai, United Arab Emirates; 7 Research Institute of Medical and Health Sciences, University of Sharjah, Sharjah, United Arab Emirates; 8 Department of Health Care Management, College of Health Sciences, University of Sharjah, Sharjah, United Arab Emirates

**Keywords:** Dubai, healthy schools, implementation, integration, joint display analysis, mixed methods, nutrition policy, public health

## Abstract

**Background:**

School Nutrition Programs (SNPs) are increasingly recognized as essential public health interventions that influence children's health, educational attainment, wellbeing, and future non-communicable disease risk. While previous studies in Dubai explored stakeholders' macro-level perceptions regarding the upscaling of SNPs and quantitatively characterized SNPs implementation across schools, the integrated understanding of how policy-level aspirations align with school-level realities remains limited. Generating such an integrated understanding is essential for informing implementation strategies that are effective, efficient, equitable, and contextually responsive.

**Objective:**

This study aimed to integrate the primary inferences generated from two complementary studies investigating SNPs in Dubai, United Arab Emirates.

**Methods:**

This study represents the third integrative phase of a multi-staged mixed methods research program. The first study qualitatively explored perceptions of key opinion leaders involved in planning, implementation, and evaluation of SNPs in Dubai. The second study quantitatively examined the status quo and characteristics of SNPs across public and private schools in Dubai. In the current study, findings from both studies were merged through a structured joint display analysis process informed by mixed methods integration approaches. Primary inferences from both datasets were iteratively compared, connected, and synthesized to generate integrated meta-inferences.

**Results:**

The integration process revealed five major meta-inferences: (1) strong policy-level commitment coexists with implementation heterogeneity across schools; (2) awareness of nutritional guidelines does not necessarily translate into comprehensive SNPs implementation; (3) implementation capacity and governance structures are key determinants of SNPs effectiveness; (4) public-private variation reflects differences in operational flexibility, resources, and institutional priorities; and (5) sustainable SNPs advancement in Dubai requires a unified systems-oriented implementation framework integrating governance, accountability, capacity building, and continuous evaluation mechanisms. Joint display analysis demonstrated convergence between stakeholder aspirations and school-level realities while also exposing critical implementation gaps.

**Conclusion:**

This integrative mixed methods analysis advances understanding of SNPs in Dubai by moving beyond triangulation toward explicit integration and meta-inference generation. Findings suggest that future SNPs advancement in Dubai should prioritize coordinated implementation governance, school-level capacity strengthening, standardized monitoring systems, and adaptive policy translation mechanisms. The generated meta-inferences provide a foundation for future policy-oriented implementation research and action.

## Introduction

School Nutrition Programs (SNPs) constitute a major pillar of child and adolescent public health promotion globally (1, 2). These programs contribute not only to nutritional wellbeing, but also to cognitive development, educational attainment, social functioning, and long-term prevention of non-communicable diseases (NCDs) (3–5). Increasingly, SNPs are also viewed as strategic societal investments capable of advancing multiple Sustainable Development Goals (SDGs), particularly those related to health, education, equity, and sustainable development (1, 6, 7).

Globally, the conceptualization of SNPs has evolved from isolated feeding initiatives toward more comprehensive ‘whole school' approaches integrating nutrition education, food environments, health promotion, family engagement, sustainability, and governance structures (1, 6).

SNPs may additionally be conceptualized within broader social determinants of health frameworks, whereby schools function as important environments shaping lifelong behavioral and health trajectories (8). Contemporary public health literature increasingly recognizes that dietary behaviors, physical activity patterns, health literacy, and future NCDs risk emerge through interactions between educational, socioeconomic, institutional, and environmental determinants rather than through individual choice alone (8, 9). Within this perspective, schools operate not merely as educational institutions, but as health-promoting ecosystems capable of influencing nutritional practices, behavioral norms, wellbeing, and health equity across the life course (8). Accordingly, the present study conceptually situates SNPs implementation within interacting macro-level policy environments and meso-level institutional implementation contexts.

Contemporary evidence suggests that the effectiveness of SNPs depends not only on the existence of nutritional policies, but also on the quality, consistency, equity, and contextual responsiveness of implementation processes (4, 10, 11). Recent implementation science literature further highlights that multi-level coordination, institutional readiness, governance mechanisms, and local operational capacity substantially influence successful policy translation into practice (11–14).

Within the Middle East and North Africa region (MENA), school-based nutrition interventions have expanded considerably over the last two decades, particularly amid increasing concern regarding childhood obesity and other NCDs (4, 5).

The relevance of SNPs within Gulf Cooperation Council (GCC) countries is amplified by ongoing epidemiological transitions characterized by increasing prevalence of obesity, sedentary lifestyles, excessive consumption of sugar-sweetened beverages, and other modifiable risk factors associated with NCDs among children and adolescents (15–17). Recent regional evidence suggests that behavioral risk patterns established during childhood and adolescence substantially contribute to the long-term burden of cardiovascular disease, diabetes, and other chronic conditions across GCC populations (17). Consequently, schools are increasingly viewed as strategic intervention settings capable of shaping healthier behavioral environments and supporting preventive public health agendas.

In the United Arab Emirates (UAE), promoting healthy lifestyles and preventing NCDs constitute national public health priorities. Consequently, schools have become important venues for health promotion initiatives targeting nutrition, physical activity, and wellbeing (3, 4).

The local relevance of this work is underscored by the UAE's epidemiological profile. National and emirate-level surveys report that approximately 30–40% of UAE schoolchildren are overweight or obese, with higher prevalence among adolescents and in private-sector schools (18, 19). These trends are paralleled by a marked nutritional transition toward energy-dense, Western-style dietary patterns, frequent consumption of sugar-sweetened beverages and ultra-processed snacks, and declining intake of traditional, plant-forward Emirati foods (15, 18). Socioeconomic determinants, including parental education, household food environment, screen-time exposure, and neighborhood food access, interact with school food environments to shape children's dietary trajectories and long-term NCD risk (8, 9, 19).

At the policy level, the UAE has progressively constructed a multi-layered SNPs architecture (20, 21). The Ministry of Health and Prevention (MoHAP) and the Emirates Authority for Standardization and Metrology issued national School Canteen Guidelines specifying permitted and prohibited food categories, while the UAE Cabinet's 2017 decision on healthy school food environments reinforced regulatory enforcement (22–24). At the emirate level, the Knowledge and Human Development Authority (KHDA) in Dubai integrates nutrition indicators within its school inspection framework, and Dubai Health Authority's wellbeing initiatives operationalize the We the UAE 2031 and National Strategy for Wellbeing 2031 mandates within educational settings (25–29). Despite this comprehensive policy scaffolding, the translation of national directives into consistent school-level practice remains uneven, a gap this study directly examines.

Despite growing policy attention, understanding of SNPs implementation in Dubai has remained fragmented. Previous work explored macro-level stakeholder perspectives regarding opportunities and challenges surrounding SNPs upscaling in Dubai (3). Subsequently, a quantitative landscape analysis examined school-level implementation characteristics across public and private schools (4). Together, these and related UAE studies have established that overweight and obesity are highly prevalent among schoolchildren with sectoral differences, that private schools' enactment of international school health standards varies considerably, and that macro-level stakeholders perceive fragmented regulatory coordination, resource constraints, and limited performance measurement as the principal barriers to upscaling. What this body of work has not established is whether macro-level policy intent and meso-level school implementation actually align, and where, specifically, they come apart. However, independently examining qualitative and quantitative findings may limit the development of deeper systemic understanding necessary for implementation advancement. Complex public health interventions such as SNPs involve interacting organizational, educational, behavioral, operational, and policy dimensions that cannot be fully understood through isolated methodological strands. Accordingly, there is increasing recognition that mixed methods research should move beyond simple triangulation toward deliberate integration processes capable of generating higher-order explanatory insights and actionable meta-inferences.

This study aimed to systematically integrate findings generated from the preceding qualitative and quantitative studies through a joint display analysis process. By merging macro-level stakeholder perspectives with meso-level school implementation realities, this study sought to generate integrated meta-inferences capable of informing future policy translation, implementation optimization, and systems-level advancement of SNPs in Dubai.

Accordingly, this study addressed the following research question:

- What meta-inferences emerge through the integration of macro-level stakeholder perceptions and school-level implementation realities pertaining to School Nutrition Programs in Dubai?
- What are the policy and practice recommendations that would enable the implementation of evidence-based SNPs across the UAE?

Through these two questions, the study deliberately adopts a policy-translation orientation, framing meta-inferences as inputs to actionable national SNPs governance and implementation reform.

## Materials and methods

### Research design

This study constitutes the third integrative phase of a multi-staged mixed methods research program investigating SNPs in Dubai, UAE. The overall research program relied on a convergent mixed methods orientation, whereby qualitative and quantitative strands were designed to complement one another and collectively contribute to systemic understanding of SNPs. Thus, the purpose of the current study was not to re-present previously published findings but to generate higher-order explanatory meta-inferences through formal mixed-methods integration.

This study is therefore best characterized as the integrative phase of a prospectively designed convergent mixed methods research program, rather than as a retrospective integration of two independently conceived studies or as a secondary analysis of existing datasets. Both strands were conducted in the same setting, on the same construct, by a substantially shared research team, under a single institutional ethical approval (MBRU-IRB-2018-029), and within overlapping data collection windows during 2019: qualitative interviews were conducted between 14 January and 18 April 2019, and the quantitative survey was administered between April and August 2019. No federal or emirate-level regulatory instrument governing school nutrition was enacted or amended between the two data collection windows; the governing instruments in force throughout were the national School Canteen Guidelines, the Dubai Municipality Guidelines and Requirements for Food and Nutrition in Schools in Dubai, and the UAE Cabinet decision of 2017. The integrated findings therefore describe a single, temporally coherent policy environment. Integration occurred at two points of interface: at the level of design, *a priori*, in that the two strands were purposefully constructed to address macro- and meso-level facets of one construct; and at the level of analysis and interpretation, following completion and independent publication of both strands, through the joint display procedure described below.

Across the research program, School Nutrition Programs were operationally defined as structured, school-based initiatives intended to shape students' nutritional intake, knowledge and food environment, encompassing any combination of seven components empirically observed across schools in Dubai: parents' involvement, lunch box provision, nutrition within the educational curriculum, hydration, awareness activities, the school canteen, and food safety and hygiene. This operationalization was applied uniformly in the quantitative instrument. The unit of analysis differed by strand: the individual key informant in the qualitative strand, and the school in the quantitative strand, reflecting the nested macro- and meso-level structure the program was designed to capture. Meta-inferences generated through integration are accordingly claims about the system, not about individual schools or individual informants.

The first phase involved a qualitative exploratory study using semi-structured interviews with key opinion leaders positioned across public health, education, and school ecosystems in the UAE (3). The second phase involved a quantitative landscape analysis examining SNPs implementation characteristics across schools in Dubai (4). Accordingly, the current study represents the integrative mixed methods analysis phase focused on merging findings from both studies through a structured joint display analysis process.

### Integration approach

Integration was conceptualized as a discrete analytic stage rather than merely a concluding triangulation exercise. The study relied on merging processes informed by contemporary mixed methods integration approaches and joint display analytic techniques (30, 31). Primary inferences generated from both preceding studies were first extracted and iteratively organized into thematic and operational domains using a hybrid inductive–deductive approach (32, 33). Deductively, domain development was informed by implementation science literature (34, 35), school health promotion frameworks (36, 37), and the overarching objectives of the mixed methods research program (31, 38). Inductively, domains were refined through repeated examination of recurring patterns, implementation-related constructs, and operational issues emerging across both qualitative stakeholder narratives and quantitative school-level findings (39, 40). Through this process, findings consistently clustered around governance and policy; implementation structures; monitoring and evaluation; stakeholder engagement; capacity building; school-level operationalization; equity and standardization; and sustainability. These domains functioned as integrative analytic categories enabling systematic comparison between macro-level and meso-level findings. Subsequently, qualitative and quantitative findings were iteratively compared through structured joint display matrices to identify convergence, complementarity, expansion, divergence, and areas of implementation tension. The iterative integration process enabled generation of higher-order meta-inferences that extended beyond the explanatory capacity of either study independently. The integration process was additionally informed by principles of methodological transparency and interpretive consistency commonly recommended in mixed methods integration literature. This two-strand integrative configuration, merging one qualitative and one quantitative study from the same research program through joint display analysis, corresponds to the canonical convergent mixed methods integration design described by Fetters, Curry, and Creswell (31) and operationalized by Guetterman, Fetters, and Creswell (41) and Haynes-Brown and Fetters (42), in which joint displays are explicitly recommended as the analytic vehicle for generating meta-inferences from paired qualitative and quantitative strands within a single research program. The complete audit trail linking each inference pair to its integration domain and integration relationship is provided in Appendix III.

### Conceptual clarification of integrative constructs

Several implementation-related constructs are used throughout the integration process and meta-inferences. For clarity, governance refers to the formal and informal arrangements through which authority, oversight, decision-making, and accountability for SNPs are exercised across stakeholders. Governance mechanisms denote the specific instruments through which governance is operationalized, including policies, reporting requirements, monitoring systems, and accountability processes. Implementation capacity refers to the organizational ability to translate policy intentions into routine practice and encompasses leadership, human resources, technical expertise, operational systems, and monitoring competencies. Resource allocation refers to the distribution of financial, human, and infrastructural resources supporting program implementation. These constructs are analytically distinct but interrelated and were used as sensitizing concepts during the integration process.

### Data sources

The integration process relied on findings generated from the following studies:

1. ‘Toward the upscaling of School Nutrition Programs in Dubai: an exploratory case-study' (3);
2. ‘School nutrition programs in Dubai: a landscape analysis' (4).

A concise summary of the design, participants, data sources, and principal findings of the two source studies is provided in Appendix II. No new primary data were collected for the current study.

Integration proceeded through five sequential steps. First, primary inferences were extracted verbatim from each published source study into a two-column matrix, preserving the original wording of each strand's conclusions. Second, each extracted inference was assigned to an analytic domain, drawing on a hybrid domain set combining deductively derived domains (from implementation science and school health promotion frameworks) with domains that emerged inductively from the extracted inferences themselves. Third, macro-level and meso-level inferences located within the same domain were compared pairwise. Fourth, each pairing was assigned an integration relationship using the typology of Fetters, Curry, and Creswell: convergence, complementarity, expansion, or divergence, with an additional category of implementation tension applied where a stated policy aspiration and an observed operational reality were neither confirmatory nor contradictory but systematically misaligned. Fifth, a meta-inference was formulated for each domain cluster, and retained only where supported by an explicitly labeled relationship identified in the preceding step. The resulting audit trail, linking each meta-inference to its contributing source inferences, analytic domain, and integration relationship, is presented in Appendix III.

### Trustworthiness and rigor

Methodological rigor was enhanced through: systematic integration procedures; iterative comparison of findings; use of structured joint display matrices; collaborative interpretation among researchers; and explicit documentation of meta-inference generation processes.

The integration process aimed to preserve methodological integrity of both qualitative and quantitative strands while maximizing interpretive depth. Domain development and meta-inference generation were conducted iteratively through collaborative review and comparison of findings across datasets. Meta-inferences were generated only when supported through explicit convergence, complementarity, or explanatory expansion across datasets. Operationally, these integration relationships were adopted from Fetters, Curry, and Creswell's typology (31): convergence denoted qualitative and quantitative findings that confirmed one another on the same construct; complementarity denoted findings that addressed different facets of the same phenomenon and together yielded a fuller picture; expansion denoted instances in which one strand extended the explanatory range of the other beyond what either could establish alone; and divergence denoted findings that were inconsistent, prompting deeper interpretive reconciliation. Three of the authors independently extracted primary inferences from both published source studies and independently produced a preliminary domain assignment before any joint discussion took place. The final domain set was established by comparing these independent assignments, retaining deductively derived domains where independently corroborated and adding inductively emergent domains where independently identified. Discrepancies in domain assignment and in relationship labeling were resolved in structured reconciliation meetings by returning to the source publications for adjudication, rather than by majority decision. The principal residual disagreement concerned the labeling of the first meta-inference, which was resolved as an implementation tension rather than as outright divergence, on the grounds that the aspiration–operation misalignment was systematic rather than contradictory; this resolution is documented transparently in Appendix I. The remaining authors then reviewed the integrated matrix and meta-inferences and provided critical input, with any disagreements resolved through discussion until consensus.

## Results

### Overview of integration outcomes

The joint display analysis process generated five overarching meta-inferences illuminating the relationship between policy-level aspirations and school-level implementation realities regarding SNPs in Dubai (Figure 1). The primary findings integrated from each source study are summarized in Appendix II and served as the evidentiary basis for the joint display analysis.

**Figure 1:**
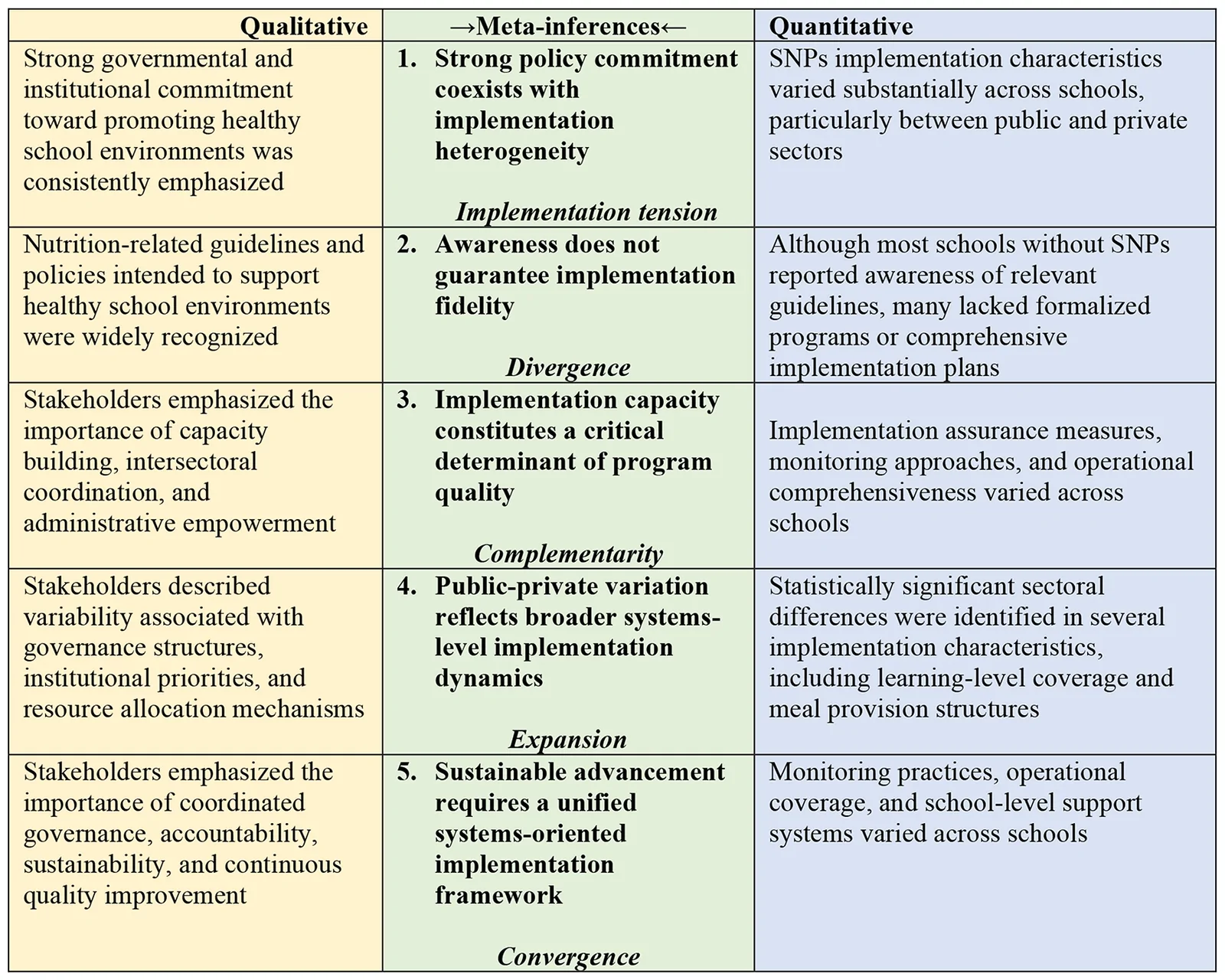
The iterative joint display analysis process generated five meta-inferences through integration of qualitative and quantitative inferences. The secondary color Green emerged by mixing the primary color Blue with the primary color Yellow (which constitutes an analogy of the lateral and critical thinking that took place to generate the meta-inferences from the integration of two sets of primary inferences). One dominant integration relationship was identified for each meta-inference (Appendix I), while recognizing that some meta-inferences also contained secondary integrative elements. Governance refers to oversight and accountability arrangements guiding SNPs implementation; implementation capacity refers to organizational competencies and resources enabling effective program delivery; systems-oriented implementation framework refers to coordinated governance, monitoring, accountability, and continuous improvement structures operating across educational and health sectors.

### Meta-inference 1: strong policy commitment coexists with implementation heterogeneity

Qualitative findings demonstrated strong governmental and institutional commitment toward promoting healthy school environments and advancing nutrition-related initiatives across Dubai. Stakeholders emphasized the strategic importance of SNPs for child wellbeing, public health, and NCD prevention.

Quantitative findings, however, revealed substantial heterogeneity in SNPs implementation characteristics across schools, particularly between public and private sectors. While most schools reported awareness of nutritional guidelines, substantial variation existed regarding implementation scope, meal coverage, operational structures, and monitoring mechanisms.

Empirically, 48 of the 60 responding schools (80.0%) reported having an SNP, yet these programs comprised markedly different combinations of the seven observed components, and 64.6% were governed primarily by internal school policy rather than by an external standard.

### Integrated interpretation

The integration suggests that policy commitment alone is insufficient to ensure standardized implementation. Translation of high-level aspirations into consistent operational realities remains dependent on contextual institutional capacities, governance mechanisms, and implementation support structures.

### Meta-inference 2: awareness does not guarantee implementation fidelity

Stakeholders highlighted the existence of nutrition-related guidelines and policies intended to support healthy school environments.

Quantitatively, although most schools without SNPs reported awareness of relevant guidelines, many still lacked formalized programs or comprehensive implementation plans.

Specifically, of the 12 responding schools without an SNP, 11 (91.7%) reported awareness of Dubai's school nutrition guidelines, and 7 (58.3%) anticipated establishing a program within three to 5 years, indicating that the constraint lies downstream of awareness.

### Integrated interpretation

The integration reveals a policy-practice gap whereby awareness and policy dissemination do not necessarily translate into implementation fidelity. Effective implementation likely requires stronger operational guidance, accountability structures, and school-level support systems.

### Meta-inference 3: implementation capacity constitutes a critical determinant of program quality

Qualitative stakeholders repeatedly emphasized the importance of capacity building, intersectoral coordination, and administrative empowerment.

Quantitative findings demonstrated variability in implementation assurance measures, monitoring approaches, and operational comprehensiveness across schools.

While 87.5% of programs had measures in place to ensure implementation, only 64.6% had defined performance indicators, and a school nutritionist was involved in just 29.2% of programs, all of them in private schools.

### Integrated interpretation

The merged findings indicate that implementation capacity represents a foundational determinant of SNPs quality and sustainability. Capacity should be conceptualized multidimensionally, encompassing: leadership competencies; operational management; monitoring literacy; nutritional expertise; and cross-sector collaboration capabilities.

### Meta-inference 4: public-private variation reflects broader systems-level implementation dynamics

Quantitative analyses identified statistically significant sectoral differences across several implementation characteristics (4). Lunch box provision differed most sharply between public and private schools (7.1% vs. 92.9%; *p* = 0.001; Cramér's V = 0.53), followed by awareness activities (19.0% vs. 81.0%; *p* = 0.047; V = 0.34), and food safety and hygiene components (20.5% vs. 79.5%; *p* = 0.048; V = 0.31). Coverage of Kindergarten (*p* = 0.008) and Primary (*p* < 0.001) learning levels was significantly more common among private schools, and every program incorporating a lunch meal was located in the private sector. The magnitude of these associations, particularly for meal provision, indicates a structural sectoral divide in implementation rather than marginal variation.

Qualitative stakeholders similarly described variability associated with governance structures, institutional priorities, and resource allocation mechanisms.

### Integrated interpretation

The integration suggests that sectoral variation reflects broader systems-level dynamics rather than isolated operational inconsistencies. Future implementation strategies should therefore standardize what an SNP must achieve, while allowing schools to vary how they achieve it.

### Meta-inference 5: sustainable advancement requires a unified systems-oriented implementation framework

Findings from both datasets emphasized the importance of coordination, accountability, sustainability, and continuous improvement.

Consistent with this, school representatives named three different external entities as responsible for their SNPs, with the distribution differing significantly by sector (Ministry of Education, *p* = 0.002; Dubai Health Authority, *p* = 0.008; Dubai Municipality, *p* = 0.049), and only 37% of programs explicitly targeted health inequalities.

### Integrated interpretation

The integration process suggests that future SNPs advancement in Dubai would benefit from a unified systems-oriented implementation framework characterized by: centralized strategic governance; context-sensitive implementation flexibility; standardized monitoring and evaluation systems; school-level capacity strengthening; family and community engagement; and iterative quality improvement mechanisms.

Such a framework may facilitate more effective, efficient, equitable, and sustainable implementation of SNPs across educational sectors.

## Discussion

This study integrated macro-level stakeholder perspectives and meso-level implementation realities pertaining to SNPs in Dubai through a structured joint display analysis process. The findings advance current understanding by generating higher-order meta-inferences that illuminate not only what exists within SNPs implementation landscapes, but also why implementation variation persists despite substantial policy commitment. To facilitate translation of the generated meta-inferences into actionable policy and practice directions, Table 1 maps each meta-inference to a corresponding implementation recommendation and responsible stakeholder group. The findings reinforce contemporary implementation science literature emphasizing that effective policy translation requires more than guideline dissemination alone (11, 13). In complex public health interventions such as SNPs, implementation outcomes are strongly shaped by organizational readiness, governance infrastructures, operational competencies, and contextual adaptation processes.

**Table 1:** Focused recommendations from each meta-inference mapped to concerned stakeholders.

No	Meta-inference	Focused recommendation	Concerned stakeholders	Basis and nature of the recommendation
1	Strong policy commitment coexists with implementation heterogeneity	Establish a unified national SNPs implementation charter that operationalizes existing policy commitments into measurable, school-level performance standards	Ministry of Health and Prevention (MoHAP); UAE Cabinet; Ministry of Education; Emirates Health Services	Directly derived; Consolidates existing instruments (new consolidating instrument, no new requirements)
2	Awareness does not guarantee implementation fidelity	Convert awareness of nutritional guidelines into mandatory implementation protocols, with structured onboarding and accountability checkpoints for school leadership	Knowledge and Human Development Authority (KHDA); ADEK; Ministry of Education; school boards and principals	Directly derived; Modifies/scales an existing instrument (KHDA inspection framework)
3	Implementation capacity is a critical determinant of program quality	Institutionalize a national SNPs capacity-building curriculum covering nutrition, monitoring literacy, project management, and intersectoral coordination for school nutrition focal points	Academic institutions [e.g., Mohammed Bin Rashid University of Medicine and Health Sciences (MBRU) – Dubai Health, United Arab Emirates University, University of Sharjah]; MoHAP training arm; school operators	Directly derived; Modifies/scales existing school staffing requirements
4	Public-private variation reflects broader systems-level dynamics	Issue sector-adapted (public vs. private) implementation playbooks anchored in a common minimum standard, with targeted support for under-resourced schools	KHDA; Abu Dhabi Department of Education and Knowledge (ADEK); private school operators; MoHAP	Directly derived; New instrument
5	Sustainable advancement requires a unified systems-oriented implementation framework	Establish a federal multi-stakeholder SNPs governance council with standardized monitoring dashboards, annual public reporting, and family/community engagement mandates	UAE Cabinet; MoHAP; Ministry of Education; KHDA; ADEK; Dubai Health; parent councils; civil society partners	Author proposition requiring validation in other emirates; New instrument

Recommendations are classified by evidentiary basis and by their relationship to existing regulatory instruments. Recommendations designated as author propositions extend beyond what the integrated evidence establishes for Dubai and require empirical validation and contextual adaptation before adoption in other emirates.

The observed implementation heterogeneity confirms, in a Gulf policy setting, what implementation science has repeatedly demonstrated elsewhere: nationally mandated health interventions in schools are delivered with substantial variability, and that variability is driven less by the content of the mandate than by the organizational conditions surrounding its delivery (43, 44). What the present integration adds is the specification of which conditions matter in Dubai. The joint display shows that where the macro-level analysis identified weakly coordinated implementation and the absence of performance measurement as system-level vulnerabilities, the meso-level analysis located those same vulnerabilities in concrete, measurable school practices—food safety and hygiene procedures (20.51% of public vs. 79.49% of private schools) and structured awareness activities (19% vs. 81%). The two strands therefore do not simply agree; the quantitative strand renders the qualitative diagnosis actionable by identifying the practices in which coordination failure becomes visible.

The sectoral pattern is the substantive contribution. In an educational ecosystem in which roughly three quarters of schools are private and fee-paying, implementation capacity is unevenly distributed by design rather than by accident: private schools operate with discretionary budgets, curriculum-linked wellbeing agendas, and inspection incentives that public schools, funded and regulated centrally, do not share in the same form. Read against the macro-level accounts of resource constraint, this suggests that the binding constraint on national SNPs performance is not the absence of standards but the absence of a mechanism that equalizes the organizational capacity to meet them.

This reading has a direct design implication. Rather than pursuing uniform delivery, policy should distinguish the core functions of SNPs—nutritional standards for food offered on premises, food safety assurance, structured nutrition education, and routine monitoring—from the forms through which schools deliver them, which may legitimately differ by sector, size, curriculum, and student population (43, 45). Such adaptive standardization preserves accountability for outcomes while permitting the contextual adaptation that the qualitative strand showed schools are already improvising in the absence of guidance.

Operationally, this argues for consolidating the currently dispersed instruments—the Ministry of Health and Prevention school canteen guidelines, the Dubai Municipality guidelines and requirements for food and nutrition in schools, the 2017 Cabinet decision on healthy food in schools, and the KHDA inspection framework - into a single school nutrition charter that specifies the core functions, assigns institutional ownership for each, and defines a small set of indicators reported through existing inspection cycles. The WHO and UNESCO global standards for health-promoting schools offer a ready template for such consolidation (46), and the integration reported here indicates what the Dubai version of that template would need to prioritize.

The findings further support growing recognition that schools should be conceptualized as complex adaptive systems rather than isolated implementation units (8, 9). This interpretation is particularly relevant in highly diverse educational ecosystems such as Dubai, where institutional heterogeneity may substantially influence implementation readiness and operational adaptation. Because school food environments interact with household and neighborhood determinants, uneven school-level implementation risks compounding, rather than mitigating, existing inequalities in children's dietary trajectories; the WHO Health Promoting Schools framework accordingly anchors family engagement as a co-equal pillar alongside school-level action (36). The WHO Health Promoting School framework for improving the health and well-being of students and their academic achievement Cochrane database of systematic reviews Cochrane database of systematic reviews (36). Consequently, future SNPs advancement efforts in Dubai may benefit from systems-oriented implementation approaches integrating governance, accountability, monitoring, adaptation, and continuous quality improvement mechanisms (47).

Importantly, the present study also contributes methodologically by demonstrating the value of explicit integration processes in mixed methods research (30, 31). Through joint display analysis, the study moved beyond parallel reporting of qualitative and quantitative findings toward generation of explanatory meta-inferences capable of informing future policy and implementation directions. The generated meta-inferences provide a strong foundation for subsequent translational and policy-oriented scholarship focused on operationalizing actionable implementation roadmaps for SNPs advancement in Dubai.

### Strengths and limitations

A major strength of this study lies in its explicit integration approach, which enabled development of systemic understanding beyond isolated methodological strands. The integration of macro-level stakeholder perceptions with school-level implementation realities generated nuanced insights with practical and policy relevance. Another strength pertains to the multi-staged design underpinning the broader research program, which facilitated examination of SNPs across multiple ecosystem levels.

The study nevertheless has limitations. Since the integration relied on previously generated datasets, no additional primary data were collected to further probe emerging meta-inferences. Additionally, findings are contextually situated within Dubai and are not generalizable to other settings. Moreover, integration across nested levels of a system cannot establish that a given macro-level perception and a given school-level observation refer to the same causal pathway; the meta-inferences should therefore be read as system-level propositions rather than as verified mechanisms. In addition, the data characterize the pre-pandemic policy configuration and warrant re-examination against current arrangements, and implementation variability could not be disaggregated by school catchment, tuition tier, curriculum, or predominant student nationality, all of which are dimensions that plausibly shape adaptation and merit dedicated investigation.

In particular, the present integration could not disaggregate implementation variability by school-level determinants such as geographic catchment, tuition tier, curriculum [e.g., American, British, International Baccalaureate (IB), Ministry of Education (MoE)], and predominant student nationality. Future primary research should explicitly model these determinants to clarify why implementation heterogeneity persists across schools and to inform equity-sensitive policy targeting.

### Implications for future research and practice

The integration of macro-level stakeholder perspectives and meso-level implementation data highlights persistent system-level variability in SNPs implementation, alongside clear convergence on the need for coordinated governance, strengthened capacity, and standardized monitoring systems. However, the current study is limited by its reliance on previously collected datasets and its cross-sectional integrative design, which constrains the ability to examine temporal dynamics of implementation change.

Future research should therefore adopt longitudinal mixed methods designs to examine how implementation capacity, governance structures, and school-level operationalization evolve over time in response to policy reforms. In particular, prospective studies could explore how adaptive implementation strategies influence reductions in heterogeneity and improvements in program fidelity across public and private schools. Future research should also further refine and test the integration framework used in this study, particularly the application of joint display methods for generating actionable meta-inferences in school health implementation research.

From an implementation perspective, future work should also operationalize and test systems-oriented frameworks identified in this study, particularly those emphasizing coordinated governance, accountability mechanisms, and continuous quality improvement. Comparative studies across different emirates or GCC contexts may further clarify how contextual factors shape implementation variability and scalability of SNPs.

## Conclusion

This study integrated findings from two complementary investigations of SNPs in Dubai through a structured joint display analysis process. The generated meta-inferences reveal that while substantial policy commitment toward healthy school environments exists in Dubai, implementation variability persists across schools due to differences in governance structures, institutional capacity, operationalization processes, and monitoring mechanisms.

The findings suggest that future advancement of SNPs in Dubai should prioritize systems-oriented implementation strategies integrating governance, accountability, capacity strengthening, continuous monitoring, and adaptive implementation support.

The findings additionally reinforce the importance of conceptualizing schools as health-promoting environments embedded within broader social, behavioral, and institutional determinants influencing child and adolescent wellbeing.

Beyond its substantive contributions, the study also demonstrates the value of deliberate mixed methods integration for generating actionable public health insights capable of informing future policy translation efforts.

While the present integration is empirically grounded in Dubai, the convergence of its meta-inferences with national MoHAP guidance and federal wellbeing strategies supports their relevance as a foundation for a unified national UAE SNPs framework, subject to confirmatory work in the other emirates.

## Data Availability

The original contributions presented in the study are included in the article/supplementary material, further inquiries can be directed to the corresponding author.
