# Supplementary material for: Integrating macro- and meso-level perspectives on school nutrition programs in Dubai: a joint display mixed methods integration study
Source: Front Public Health. 2026 Oct 6;14:1915072. doi: 10.3389/fpubh.2026.1915072 (PMC13680410; doi:10.3389/fpubh.2026.1915072)
Supplement: Supplementary file 1 [file Supplementary_File_1.docx]

## Appendix I. ‘Best fit’ integration relationship vis-à-vis rationale

| **Meta-inference** | **Dominant integration relationship*** | **Rationale** |
| --- | --- | --- |
| **1. Strong policy commitment coexists with implementation heterogeneity** | **Implementation tension** | The qualitative data show strong commitment, while quantitative findings show inconsistent implementation. This is not outright contradiction, but a tension between aspirations and operational realities. |
| **2. Awareness does not guarantee implementation fidelity** | **Divergence** | Awareness/policy recognition does not align with actual implementation. The datasets point in different directions regarding policy uptake versus practice. |
| **3. Implementation capacity constitutes a critical determinant of program quality** | **Complementarity** | The qualitative findings explain why variability exists, while the quantitative findings demonstrate where/how variability appears operationally. The datasets enrich one another. |
| **4. Public-private variation reflects broader systems-level implementation dynamics** | **Expansion** | The quantitative strand identifies sectoral differences, while the qualitative strand broadens interpretation by situating those differences within governance and institutional contexts. |
| **5. Sustainable advancement requires a unified systems-oriented implementation framework** | **Convergence** | Both datasets independently support the need for coordination, accountability, monitoring, and sustainability mechanisms. |

*In joint displays, it is standard to classify by the dominant integrative relationship. Yet, meta-inference 1 also contains divergence; meta-inference 4 also contains complementarity; and meta-inference 5 also contains complementarity.

## Appendix II. Summary of source studies included in the integration

| **Attribute** | **Phase I - Qualitative** | **Phase II - Quantitative** |
| --- | --- | --- |
| **Design** | Exploratory qualitative study, semi-structured key informant interviews | Cross-sectional landscape analysis, tailor-made structured survey |
| **Level of inquiry** | Macro (system, emirate, institutional leadership) | Meso (school) |
| **Sampling** | Purposive with snowball augmentation, across regulatory, health, education and school leadership roles | Stratified probabilistic sampling of 75 schools (21 public, 54 private) from 280 schools in Dubai; Cochran-derived requirement of 59 for 95% confidence |
| **Participants** | 48 key informants invited; 29 participated (60%); interviews continued to saturation | 60 schools responded (overall response rate 0.80; public 19/21 = 0.90; private 41/54 = 0.76); 48 (80%) had an SNP (13 public, 35 private) |
| **Data collection** | 14 January - 18 April 2019; semi-structured interview guide; audio-recorded and transcribed verbatim | April - August 2019; content- and face-validated structured instrument completed by a nominated school representative following telephone contact and email distribution |
| **Analysis** | Framework-based thematic analysis (five-step approach), using a health systems model as the analytic framework; independent coding with reconciliation | Descriptive proportions; chi-square tests of independence for sectoral comparisons; SPSS v27 |
| **Ethics** | MBRU-IRB-2018-029 | MBRU-IRB-2018-029 (same approval) |
| **Principal findings** | Five themes: (i) fragmented coordination across federal and local regulators with a multiplicity of guidelines; (ii) human and financial resource constraints; (iii) weakly coordinated implementation efforts; (iv) absence of systematic performance measurement; (v) need for greater inclusiveness given a student body of more than 200 nationalities | SNP prevalence 80.0%; 64.6% governed by internal school policy; significant sectoral differences in lunch box provision (p = 0.001, V = 0.53), awareness activities (p = 0.047, V = 0.34) and food safety/hygiene (p = 0.048, V = 0.31); implementation assurance measures in 87.5% but defined performance indicators in only 64.6%; nutritionist involvement in 29.2% (all private); 37% targeted health inequalities; among the 12 schools without SNPs, 91.7% aware of guidelines and 58.3% planning a program within 3-5 years |

## Appendix III. Integration audit trail

This appendix documents, for each meta-inference, the specific qualitative inference and quantitative result that were brought into dialogue, the integration domain under which they were compared, and the integration relationship (confirmation, expansion, or discordance) that was assigned. It is provided so that the analytic path from single-study findings to joint-display meta-inferences is fully traceable.

| **Meta-inference** | **Contributing macro-level inferences (Phase I)** | **Contributing meso-level findings (Phase II)** | **Analytic domain** | **Integration relationship** |
| --- | --- | --- | --- | --- |
| MI 1 - Policy aspiration exceeds implementation consistency | Fragmented coordination across federal and local regulators; weakly coordinated implementation efforts | SNP prevalence 80.0%; heterogeneous composition across the seven components; 64.6% governed by internal school policy | Policy–practice alignment | Implementation tension |
| MI 2 - Awareness does not guarantee implementation fidelity | Existence and dissemination of nutrition guidelines and policies | Among 12 schools without SNPs: 91.7% aware of guidelines; 58.3% planning a program in 3–5 years | Guideline dissemination and uptake | Divergence |
| MI 3 -Implementation capacity determines program quality | Human and financial resource constraints; need for capacity building and administrative empowerment | Implementation assurance measures 87.5% but defined performance indicators 64.6%; nutritionist involvement 29.2% (all private); medical staff 70.8% (p = 0.034) | Institutional capacity and workforce | Complementarity |
| MI 4 - Public–private variation reflects systems-level dynamics | Variability attributed to governance structures, institutional priorities and resource allocation | Lunch box p = 0.001, V = 0.53; awareness activities p = 0.047, V = 0.34; food safety/hygiene p = 0.048, V = 0.31; all lunch-providing SNPs private; KG p = 0.008, Primary p < 0.001 | Governance autonomy and sectoral structure | Expansion |
| MI 5 - Systemic governance and monitoring are the binding constraint | Absence of systematic performance measurement; fragmented regulatory coordination; need for inclusiveness across 200+ nationalities | Responsibility attributed variously to MoE (p = 0.002), DHA (p = 0.008), Municipality (p = 0.049); defined performance indicators 64.6%; 37% targeted health inequalities | Governance, accountability and monitoring | Convergence |
